# Optimization of Gradient Descent Parameters in Attitude Estimation Algorithms

**DOI:** 10.3390/s23042298

**Published:** 2023-02-18

**Authors:** Karla Sever, Leonardo Max Golušin, Josip Lončar

**Affiliations:** Department of Communication and Space Technologies, Faculty of Electrical Engineering and Computing, University of Zagreb, Unska 3, 10000 Zagreb, Croatia

**Keywords:** attitude estimation, rotational quaternion, Euler angles, gradient descent algorithm, complementary filter, optimization

## Abstract

Attitude estimation methods provide modern consumer, industrial, and space systems with an estimate of a body orientation based on noisy sensor measurements. The gradient descent algorithm is one of the most recent methods for optimal attitude estimation, whose iterative nature demands adequate adjustment of the algorithm parameters, which is often overlooked in the literature. Here, we present the effects of the step size, the maximum number of iterations, and the initial quaternion, as well as different propagation methods on the quality of the estimation in noiseless and noisy conditions. A novel figure of merit and termination criterion that defines the algorithm’s accuracy is proposed. Furthermore, the guidelines for selecting the optimal set of parameters in order to achieve the highest accuracy of the estimate using the fewest iterations are proposed and verified in simulations and experimentally based on the measurements acquired from an in-house developed model of a satellite attitude determination and control system. The proposed attitude estimation method based on the gradient descent algorithm and complementary filter automatically adjusts the number of iterations with the average below 0.5, reducing the demand on the processing power and energy consumption and causing it to be suitable for low-power applications.

## 1. Introduction

The attitude estimation of an object has a significant role in many different areas of technology: from consumer electronics [1] and human motion analysis [2,3] to robotics [4] and aerospace technology [5,6,7,8]. For each of these technologies, precise and accurate attitude measurements are of great importance. Specifically, the choice of sensors and advanced processing techniques determines the precision and accuracy. In general, the attitude is expressed as the rotation between the Earth’s inertial frame and the body’s local frame. This rotation in 3D space can be expressed by using Euler angles [9], or more commonly by using quaternions [10,11]. Although less intuitive, quaternions avoid the singularity of Euler angles known as the gimbal lock [12], which is the main reason of quaternion supremacy over Euler angles. The attitude estimation algorithms are based on a mathematical description of a body frame rotation with respect to a global, usually inertial, frame. In the literature, various direct attitude estimation methods have been reported, such as the q-method [13], Estimator of Quaternion (ESOQ) [14], Quaternion Estimator (QUEST) [15], and Recursive Quaternion Estimator (REQUEST and Optimal-REQUEST) [16,17]. Since attitude estimation is necessarily based on multi-sensor observations, numerous sensor fusion techniques have been developed. These techniques combine measurements from various different sensors into a single, more accurate, and reliable attitude estimate. Often, an integrated inertial measurement unit (IMU) consisting of a tri-axis accelerometer, a tri-axis magnetometer, and a tri-axis gyroscope provides a low cost, lightweight, and easily accessible measurement system [18,19,20,21]. Although easily accessible, these sensors have some major drawbacks that complicate the attitude estimation. Accelerometers are used as the devices for measuring gravitational acceleration, which points directly toward the center of Earth. However, other than gravitational acceleration, accelerometers measure any acceleration caused by forces acting on the sensor. On the other hand, magnetometers suffer from the influence of hard and soft iron effects caused by different magnetic materials present in the vicinity of the sensor interfering with the measurement and calibration. In terms of the noise level, gyroscopes show the highest quality of the three. They provide information about the angular velocity of an object and require numerical integration for attitude estimation that results in very low noise levels. Nevertheless, they suffer from an error accumulation caused by the ever-present bias. Although IMU sensors cannot provide reliable attitude estimation on their own, through sensor fusion techniques, adequate accuracy can be attained. By far the most widespread sensor fusion technique for attitude estimation used in practice is the Kalman filter [19,20,21] due to its high accuracy and efficiency. Despite the advantages of the Kalman filter, its implementation is more complex and requires higher computational power, which may not be suitable for low-power applications. In systems where the usage of a Kalman filter is neither possible nor preferable, less-complex sensor fusion techniques such as a complementary filter may be utilized [22,23,24,25]. Complementary filters offer an efficient, computationally inexpensive, and accurate attitude estimate by combining high-pass filtered gyroscope attitude estimate with the low-pass filtered accelerometer and a magnetometer attitude estimate. As mentioned above, there are many direct methods for attitude estimation that could be used for attitude estimation using an accelerometer and magnetometer. In this paper, we focus on one of the most recent iterative techniques based on the gradient descent algorithm [22,26,27,28,29,30].

The gradient descent algorithm combines accelerometer and magnetometer measurements to estimate the optimal rotational quaternion in terms of the least square error. Since the algorithm is iterative, the required number of iterations is quite an important parameter that ultimately affects the accuracy and processing time. Too few or too many iterations of the gradient descent algorithm indicate a suboptimal set of gradient descent parameters, which leads to either the inaccurate estimation of the rotational quaternion or wasting computational resources, as it will be detailed throughout the paper. Thus, the number of iterations is crucial in real time applications. However, this is often overlooked or completely ignored in the literature. Some reports state that a single iteration of the gradient descent algorithm is sufficient for accurate attitude estimation provided that the convergence rate of the estimated orientation is equal or greater than the rate of change in the object rotation [30]. However, the number of iterations depends on many parameters and is difficult to predict. Thus, in this paper, we propose an optimization method of the gradient descent parameters applied to the estimation of the rotational quaternion. We propose a novel figure of merit and the criterion based on which the iterative process is terminated. The termination criterion allows for an automatic determination of the required number of iterations. Using this approach, we show that the average number of iterations of less than one can be achieved. Furthermore, we present the analysis of three methods for the initial quaternion propagation, noise analysis, and experimental verification.

The paper is organized as follows. Section 2 describes the attitude representation based on the Euler angles and rotational quaternion, as well as the relation between them. Section 3 brings the mathematical background behind the attitude estimation based on the gradient descent algorithm and complementary filter. Section 4 provides a detailed description of the optimization of each gradient descent parameter. Section 5 describes the experimental results and observations, while Section 6 brings final conclusions and guidelines for future work.

## 2. Attitude Representation

**Figure 1:**
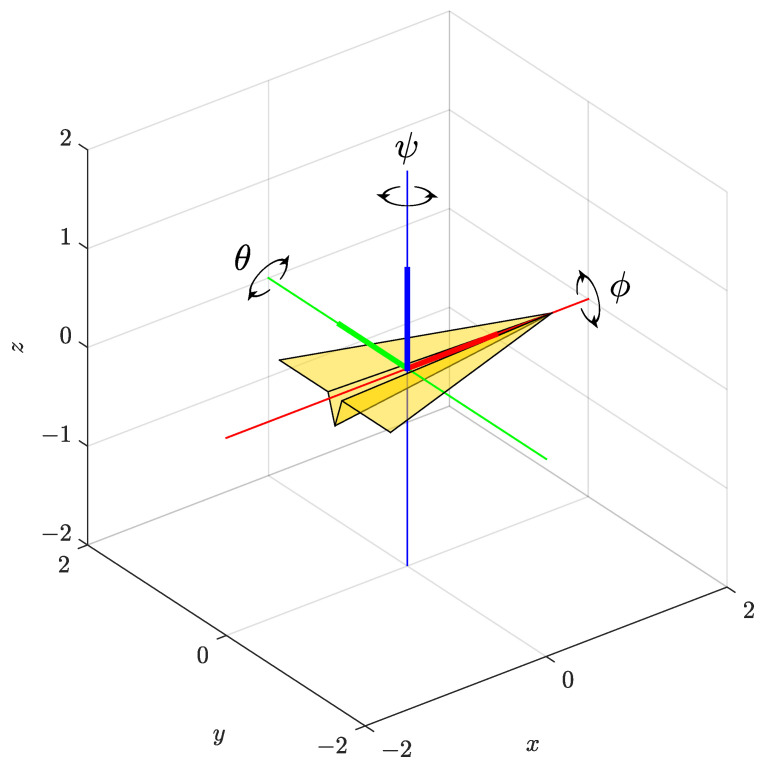
Euler angles ψ, θ, and ϕ representing the attitude of an object in 3D space.

The attitude of a body can be expressed as a rotation between the coordinate system of a body relative to the inertial, most often Earth’s, coordinate system. A simple and intuitive representation of an object’s orientation in three-dimensional (3D) space can be obtained from Euler angles—yaw (ψ), pitch (θ), and roll (ϕ), which represent rotations of a body around three orthogonal axes: *z*-, *y*-, and *x*-axis, respectively, as shown in Figure 1. Each of the three mentioned rotations can be expressed in the form of a rotation matrix. Multiplying the three matrices yields a single rotation matrix known as the direction cosine matrix (DCM). Here, it should be noted that the Euler rotational sequence is important due to the the fact that matrix multiplication is not commutative. In the following text, a conventional z−y−x rotation sequence for aerospace applications will be used.

The first rotation is applied around the *z*-axis of the inertial coordinate system for angle ψ. This rotation defines a new local coordinate system $V_{1}$ whose *z*-axis is aligned with the *z*-axis of the inertial coordinate system, while the *x* and *y*-axis of the coordinate system $V_{1}$ are rotated for angle ψ. This rotation is represented by the matrix $R_{i}^{v_{1}}\left(\psi \right)$.

(1) $$R_{i}^{v_{1}}\left(\psi \right)=\left[\begin{array}{ccc} \cos(\psi ) & \sin(\psi ) & 0\\ -\sin(\psi ) & \cos(\psi ) & 0\\ 0 & 0 & 1 \end{array}\right]$$

Similarly, matrix $R_{v_{1}}^{v_{2}}\left(\theta \right)$ represents the rotation around *y*-axis of the coordinate system $V_{1}$ (defined by the previous rotation) for angle θ. The rotation creates a new local coordinate system $V_{2}$ whose *y*-axis is aligned with the *y*-axis of the coordinate system $V_{1}$, while the *x* and *z*-axis of the coordinate system $V_{2}$ are rotated for angle θ with respect to the coordinate system $V_{1}$.

(2) $$R_{v_{1}}^{v_{2}}\left(\theta \right)=\left[\begin{array}{ccc} \cos(\theta ) & 0 & -\sin(\theta )\\ 0 & 1 & 0\\ \sin(\theta ) & 0 & \cos(\theta ) \end{array}\right]$$

Finally, matrix $R_{v_{2}}^{b}\left(\phi \right)$ defines a rotation around the *x*-axis of the coordinate system $V_{2}$ for angle ϕ. Again, the rotation creates a new, body coordinate system *B* whose *x*-axis is aligned with the *x*-axis of the $V_{2}$ coordinate system, while the *y* and *z*-axis of the coordinate system *B* are rotated for angle ϕ with respect to the coordinate system $V_{2}$.

(3) $$R_{v_{2}}^{b}\left(\phi \right)=\left[\begin{array}{ccc} 1 & 0 & 0\\ 0 & \cos(\phi ) & \sin(\phi )\\ 0 & -\sin(\phi ) & \cos(\phi ) \end{array}\right]$$

To obtain the DCM matrix, the rotation matrices defined by (1)–(3) are multiplied respecting the above-presented z−y−x sequence.

(4) $$R_{i}^{b}\left(\phi ,\theta ,\psi \right)=R_{v_{2}}^{b}\left(\phi \right)R_{v_{1}}^{v_{2}}\left(\theta \right)R_{i}^{v_{1}}\left(\psi \right)$$

The DCM elements can be expressed as functions of Euler angles ψ, θ, and ϕ as follows:

(5) $$R_{i}^{b}\left(\phi ,\theta ,\psi \right)=\left[\begin{array}{ccc} \cos(\psi )\cos(\theta ) & \cos(\theta )\sin(\psi ) & -\sin(\theta )\\ \cos(\psi )\sin(\phi )\sin(\theta )-\cos(\phi )\sin(\psi ) & \cos(\phi )\cos(\psi )+\sin(\phi )\sin(\psi )\sin(\theta ) & \cos(\theta )\sin(\phi )\\ \sin(\phi )\sin(\psi )+\cos(\phi )\cos(\psi )\sin(\theta ) & \cos(\phi )\sin(\psi )\sin(\theta )-\cos(\psi )\sin(\phi ) & \cos(\phi )\cos(\theta ) \end{array}\right].$$

Despite being simple and intuitive, the attitude representation based on Euler angles is not preferred in many applications due to the effect known as gimbal lock, a singularity of Euler angles that manifests itself in the loss of a rotational degree of freedom. When using Euler angles for attitude representation, the gimbal lock is present regardless of the chosen rotational sequence. For the z−y−x rotation sequence, the gimbal lock occurs for $\theta =\pm 90^{\circ }$.

To avoid this problem, an alternative mathematical description of body orientation can be used. One of the most common and practical alternatives for Euler angles are rotational quaternions. The rotational quaternions are generalized complex numbers consisting of one real and three imaginary, mutually orthogonal units, which can likewise represent the rotation between the two coordinate systems. Unlike Euler angles, which depend on the rotation sequence, the rotational quaternions can be described by a single angle of rotation α around the unit vector $n=n_{x}\hat{i}+n_{y}\hat{j}+n_{z}\hat{k}$, representing the axis of rotation ($\hat{i}$, $\hat{j}$, and $\hat{k}$ are basis vectors of the inertial Cartesian coordinate system).

(6) $$n=\sqrt{n_{x}^{2}+n_{y}^{2}+n_{z}^{2}}=1$$

The rotational quaternion can be represented by the four-dimensional (4D) vector defined as follows:

(7) $$q=\cos\left(\frac{\alpha }{2}\right)+\sin\left(\frac{\alpha }{2}\right)n=\left[\begin{array}{c} q_{1}\\ q_{2}\\ q_{3}\\ q_{4} \end{array}\right]=\left[\begin{array}{c} \cos(\alpha /2)\\ n_{x}\sin\left(\alpha /2\right)\\ n_{y}\sin\left(\alpha /2\right)\\ n_{z}\sin\left(\alpha /2\right) \end{array}\right].$$

Similar to (5), the elements of DCM can be expressed as functions of quaternion elements.

(8) $$R_{i}^{b}\left(q\right)=\left[\begin{array}{ccc} q_{1}^{2}+q_{2}^{2}-q_{3}^{2}-q_{4}^{2} & 2(q_{2}q_{3}+q_{1}q_{4}) & 2(q_{2}q_{4}-q_{1}q_{3})\\ 2(q_{2}q_{3}-q_{1}q_{4}) & q_{1}^{2}-q_{2}^{2}+q_{3}^{2}-q_{4}^{2} & 2(q_{3}q_{4}+q_{1}q_{2})\\ 2(q_{2}q_{4}+q_{1}q_{3}) & 2(q_{3}q_{4}-q_{1}q_{2}) & q_{1}^{2}-q_{2}^{2}-q_{3}^{2}+q_{4}^{2} \end{array}\right]$$

Using (5) and (8), a direct relation between Euler angles and the rotational quaternion can be found. Thus, the Euler angles expressed as functions of rotation quaternion can be written as:

(9) $$\left[\begin{array}{c} \psi \\ \theta \\ \phi \end{array}\right]=\left[\begin{array}{c} \arctan\left(\frac{r_{12}}{r_{11}}\right)\\ -\arcsin(r_{13})\\ \arctan\left(\frac{r_{23}}{r_{33}}\right) \end{array}\right]=\left[\begin{array}{c} \arctan\left(\frac{2(q_{2}q_{3}+q_{1}q_{4})}{q_{1}^{2}+q_{2}^{2}-q_{3}^{2}-q_{4}^{2}}\right)\\ -\arcsin(2\left(q_{2}q_{4}-q_{1}q_{3}\right))\\ \arctan\left(\frac{2(q_{3}q_{4}+q_{1}q_{2})}{q_{1}^{2}-q_{2}^{2}-q_{3}^{2}+q_{4}^{2}}\right) \end{array}\right].$$

## 3. Attitude Estimation Methods Based on Gradient Descent Algorithm

### 3.1. Attitude Estimation Based on Gradient Descent Algorithm

The gradient descent algorithm is an iterative algorithm often used in optimization problems for finding the local minimum of an arbitrary cost function *J*. Applied to the problem of attitude estimation, the cost function is simply a function of the rotational quaternion. The gradient descent can be defined by a simple recurrence relation:

(10) $$q_{k+1}=q_{k}-\mu \nabla J\left(q_{k}\right),$$

where q is the rotational quaternion and μ is a step size that defines the convergence rate. The algorithm starts by assuming an initial quaternion $q_{0}$ that is updated for each *k* as the cost function $J(q_{k})$ approaches its minimum. The smaller the μ, the longer it requires for the algorithm to converge. On the other hand, if μ is too large, the algorithm may diverge and the minimum of the cost function may not be reached. Therefore, μ is often experimentally determined. Since our goal is to find the rotational quaternion that optimally represents the attitude of a body, the cost function being minimized is $J\left(q\right):R^{4}\to R$. To define the cost function, let us first define the error vectors.

Let the vector $a^{b}$ be the body frame gravitational acceleration vector and let $R_{i}^{b}$ be the rotation matrix that describes the relation between the inertial and body reference frames. Ideally, if $R_{i}^{b}$ is a function of the correct rotation quaternion, the vector $a^{b}$ can be calculated as $a^{b}=R_{i}^{b}a^{i}$, with $a^{i}$ being the referent vector of gravitational acceleration expressed in the inertial frame of reference. If the rotational quaternion defining the matrix $R_{i}^{b}$ does not correctly describe the attitude of a body, the error vector $e_{a}$ can be calculated as:

(11) $$e_{a}=R_{i}^{b}a^{i}-a^{b}.$$

The error vector $e_{b}$, which describes the rotational error of the magnetic field vector, is calculated in a similar manner.

(12) $$e_{m}=R_{i}^{b}m^{i}-m^{b}$$

Vectors $a^{i}$ and $m^{i}$ represent known referent vectors of the gravitational acceleration and Earth’s magnetic field, respectively, represented in the inertial frame of reference. Using (11) and (12), we define the cost function J(q) as follows:

(13) $$J\left(q\right)=e_{a}^{T}e_{a}+e_{m}^{T}e_{m}.$$

Notice that, in ideal lossless conditions, the value of the cost function is equal to zero for the rotational quaternion representing the true attitude of an object, since both error vectors are equal to zero vectors. In reality, however, this is not the case due to unavoidable noise and sensor imperfections. Although these imperfections cannot be eliminated, the value of the cost function can be minimized using the gradient descent algorithm. In order to apply the gradient descent algorithm, one needs to derive the expression for the gradient of the cost function. To do so, the partial derivatives of the cost function (13) with respect to the *i*-th element of the rotation quaternion are derived.

(14) $$\frac{\partial }{\partial q_{i}}J\left(q\right)=2e_{a}^{T}\left(\frac{\partial }{\partial q_{i}}R_{i}^{b}\right)a^{i}+2e_{m}^{T}\left(\frac{\partial }{\partial q_{i}}R_{i}^{b}\right)m^{i}$$

Here, $\partial R_{i}^{b}/\partial q_{i}$ represents the partial derivative of the rotation matrix $R_{i}^{b}$ (8), with respect to the *i*-th element of the rotation quaternion, as provided below.

(15a) $$M_{1}=\frac{\partial }{\partial q_{1}}R_{i}^{b}=2\left[\begin{array}{ccc} q_{1} & q_{4} & -q_{3}\\ -q_{4} & q_{1} & q_{2}\\ q_{3} & -q_{2} & q_{1} \end{array}\right]$$

(15b) $$M_{2}=\frac{\partial }{\partial q_{2}}R_{i}^{b}=2\left[\begin{array}{ccc} q_{2} & q_{3} & q_{4}\\ q_{3} & -q_{2} & q_{1}\\ q_{4} & -q_{1} & -q_{2} \end{array}\right]$$

(15c) $$M_{3}=\frac{\partial }{\partial q_{3}}R_{i}^{b}=2\left[\begin{array}{ccc} -q_{3} & q_{2} & -q_{1}\\ q_{2} & q_{3} & q_{4}\\ q_{1} & q_{4} & -q_{3} \end{array}\right]$$

(15d) $$M_{4}=\frac{\partial }{\partial q_{4}}R_{i}^{b}=2\left[\begin{array}{ccc} -q_{4} & q_{1} & q_{2}\\ -q_{1} & -q_{4} & q_{3}\\ q_{2} & q_{3} & q_{4} \end{array}\right]$$

Finally, using (14) and (15), one defines the gradient of the cost function at $q_{k}$.

(16) $$\nabla J\left(q_{k}\right)=2\left[\begin{array}{c} e_{a}^{T}M_{1}a^{i}\left(q_{k}\right)+e_{m}^{T}M_{1}m^{i}\left(q_{k}\right)\\ e_{a}^{T}M_{2}a^{i}\left(q_{k}\right)+e_{m}^{T}M_{2}m^{i}\left(q_{k}\right)\\ e_{a}^{T}M_{3}a^{i}\left(q_{k}\right)+e_{m}^{T}M_{3}m^{i}\left(q_{k}\right)\\ e_{a}^{T}M_{4}a^{i}\left(q_{k}\right)+e_{m}^{T}M_{4}m^{i}\left(q_{k}\right) \end{array}\right]$$

The performance of the gradient descent algorithm in attitude estimation depends on a number of parameters, such as the step size μ, maximum number of iterations $N_{max}$, and initial quaternion $q_{0}$, investigated in the following sections. Moreover, special care is devoted to defining the termination criterion and investigating different methods of initial quaternion propagation. To optimize the performance of the gradient descent algorithm, one should choose the optimal set of parameters that yields the highest accuracy of the estimates obtained in the fewest number of iterations.

### 3.2. Complementary Filter Based on Gradient Descent Algorithm

A complementary filter is a common approach for the fusion of different sensor types. It uses a low-pass and a high-pass filter with the same cutoff frequency to maintain the advantages and reject the disadvantages of each sensor type. The implementation of the complementary filter is very straightforward and lightweight, which is why it is often preferred in the low-power applications. The complementary filter combines the estimates obtained using different sensors and techniques using a simple weighted sum. For example, the rotation quaternion can be estimated by using an accelerometer and a magnetometer through the gradient descent algorithm as described above. Let us denote this estimated quaternion with $q_{\mathrm{gd}}$. Likewise, it can also be estimated based on the angular velocity measured using a gyroscope. This estimate is denoted by $q_{\omega }$. The weighted sum, representing the implementation of the complementary filter, can be written simply as:

(17) $$q=Kq_{\omega }+\left(1-K\right)q_{\mathrm{gd}}.$$

Here, *K* represents scaling factor (also known as the complementary filter gain) and q the final estimate of the rotation quaternion. Since a gyroscope is a rate sensor (i.e., it provides the information about the angular velocity ω of an object), $q_{\omega }$ is estimated through the process of numerical integration as follows:

(18) $$q_{k}=q_{k-1}+\Delta T{\dot{q}}_{k},$$

where ΔT is the time between two samples and ${\dot{q}}_{k}$ is the rate of change (i.e., the time derivative) of the rotation quaternion in step *k*. $\dot{q}$ can be calculated from the angular velocity as follows [31]:

(19) $$\dot{q}=\frac{d}{dt}q=\frac{1}{2}\Omega \left(\omega \right)q.$$

Here, the matrix Ω(ω) is defined as [31]:

(20) $$\Omega \left(\omega \right)=\left[\begin{array}{cccc} 0 & -\omega_{x} & -\omega_{y} & -\omega_{z}\\ \omega_{x} & 0 & \omega_{z} & -\omega_{y}\\ \omega_{y} & -\omega_{z} & 0 & \omega_{x}\\ \omega_{z} & \omega_{y} & -\omega_{x} & 0 \end{array}\right].$$

While the process of numerical integration highly reduces the measured noise, integrating the bias present in gyroscopes leads to the inevitable drift of the estimated rotational quaternion. Thus, $q_{\omega }$ is high-pass filtered through the complementary filter. Moreover, gyroscopes as the rate sensors do not provide information about the absolute attitude of an object. Here, the absolute attitude is provided by the acceleromater and magnetometer. These sensors are much more affected by noise. Thus, $q_{\mathrm{gd}}$ is low-pass filtered through the complementary filter. Consequently, the final estimate inherits the advantages of each estimation method, while the summation of the rotational quaternion in general does not maintain the unit length of the quaternion, it is applicable to the quaternionions close to each other in the quaternion space. Nevertheless, it is recommended to normalize the final estimated quaternion to enforce the unit length and preserve the convexity of the estimation problem [32].

## 4. Simulations and Optimization of Gradient Descent Parameters

As mentioned in the previous sections, there are several parameters that determine the performance of the gradient descent algorithm in the estimation of the rotational quaternion. These parameters are the step size μ, maximum allowed number of iterations $N_{max}$, and initial rotational quaternion $q_{0}$. In this section, we analyze the effect of these parameters on the number of iterations and principal angle error (PAE) of the estimate. Moreover, we present and evaluate different methods of initial quaternion propagation and introduce yet another parameter ($G_{max}$) as the figure of merit and termination criterion. The goal is to present the methodology and guidelines for selecting the optimal set of parameters that provides the highest accuracy of the estimate using the fewest iterations, which represent the opposing requirements in iterative algorithms. Finding the optimal parameter set leads to lower power consumption and lowers the required computational power, which may be of crucial importance, especially in low-power applications.

The simulations and results presented in this section are based on the software generated three-axes gravitational acceleration, three-axes magnetic field, and three-axes angular velocity characteristic for IMUs. In these simulations, the referent vector of gravitational acceleration is chosen to be $a^{i}={\left[\begin{array}{ccc} 0 & 0 & 9.81 \end{array}\right]}^{T}$ m/s${}^{2}$, and the referent vector of the magnetic field $m^{i}={\left[\begin{array}{ccc} 22.2 & 1.7 & 42.7 \end{array}\right]}^{T}$ μT, according to the World Magnetic Model [33] at the location 45${}^{\circ }$48${}^{\prime }$26${}^{\prime \prime }$ N, 15${}^{\circ }$58${}^{\prime }$3${}^{\prime \prime }$ E, representing the coordinates of Zagreb, Croatia. The software generated data are based on the ground truth rotational quaternions known for every sample, which allow for the calculation of the PAE. The PAE is extracted from the corrective rotational matrix defined as $R^{T}\hat{R}$, R, and $\hat{R}$ being the true and estimated rotational matrices, respectively. For each sample, the PAE is calculated as:

(21) $$PAE=\arccos\left(\frac{1}{2}\left(\mathrm{Tr}(R^{T}\hat{R})-1\right)\right).$$

The simulations are performed in an ideal, noiseless condition as well as with the Gaussian white noise added to the software generated data. The noise levels of all three sensors (accelerometer, magnetometer, and gyroscope) correspond to the noise levels of the low cost IMU MPU9250 used as a reference. Before the noise analysis, the magnetometer calibration procedure was performed. To eliminate the unwanted artifacts, the noise levels were extracted from the measurement of more than 3500 samples with the IMU in static position. The noise variance applied to the software generated data is the highest variance extracted from the measurement for each sensor, namely, $0.2\times 10^{-3}$ (m/s${}^{2}$)${}^{2}$ for the accelerometer, 0.65 (μT)${}^{2}$ for the meagnetometer, and $0.35\times 10^{-6}$ (rad/s)${}^{2}$ for the gyroscope.

### 4.1. Selection of Step Size

The step size, denoted by μ in (10), is one of the most important parameters in the gradient descent algorithm. It is a positive real number that determines how fast the estimate converges toward the ground truth. If not chosen carefully, it may cause divergence of the algorithm. The step size is often empirically determined. In order to select the optimal step size, let us assume that the attitude of an object is represented by the rotational quaternion q defined by the arbitrarily chosen vector of rotation $n={\left[\begin{array}{ccc} 1 & -1 & 0 \end{array}\right]}^{T}$ and the angle of rotation α. Based on (7), for the given angle α, the true rotational quaternion q can be calculated. Note that the vector of rotation n should be normalized before calculating the rotational quaternion. Using (8), the rotation matrix $R_{i}^{b}\left(q\right)$ can be calculated, which, together with the known reference vectors of the gravitational acceleration $a^{i}$ and the magnetic field $m^{i}$ expressed in the inertial frame of reference, were used to calculate the representation of the same physical quantities in the object’s body frame ($a^{b}=R_{i}^{b}a^{i}$ and $m^{b}=R_{i}^{b}m^{i}$).

**Figure 2:**
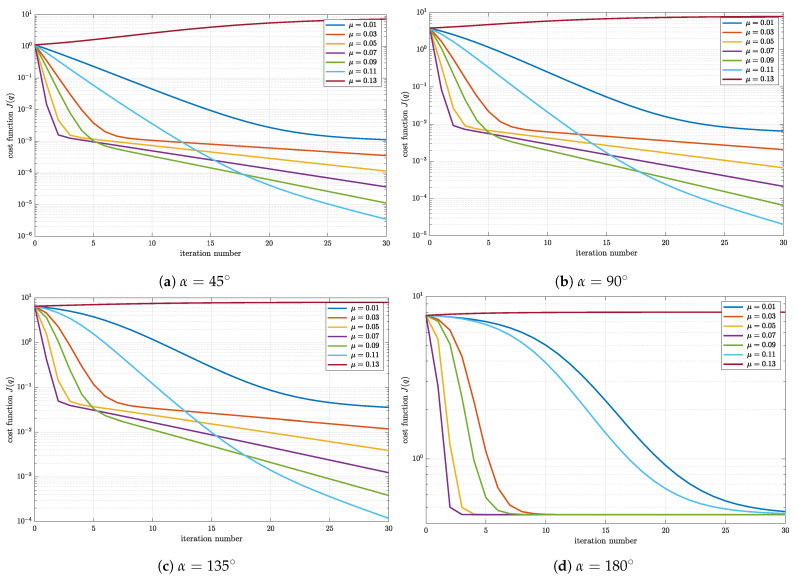
Cost function J(q) of each iteration for different step size μ and angle of rotation α.

In this simulation, no noise was added to the calculated body frame vectors. The body frame vectors were passed as the inputs to the gradient descent algorithm implemented in MathWorks Matlab as described in the previous section. The initial rotational quaternion was chosen to be $q_{0}={\left[\begin{array}{cccc} 1 & 0 & 0 & 0 \end{array}\right]}^{T}$. The value of the cost function J(q) for each iteration of the gradient descent algorithm is plotted in Figure 2 for different values of step size μ and angle α.

Focusing on Figure 2a plotted for the rotation angle $\alpha =45^{\circ }$, one can observe that the convergence rate of the cost function highly depends on the step size value. The values of tested step sizes are chosen in the range from 0.01 to 0.13. The initial value of the cost function is equal for each step size. As expected, the value of the cost function drops with each iteration indicating the drop of error in estimated rotational quaternion (13), except for μ=0.13. This is the largest step size in the range and causes the divergence of the gradient descent, which manifests in monotonic growth of the cost function with each iteration. For the other values of the step size, a clear difference in steepness is observable. For all step sizes (except μ=0.13), the knee in each graph that represents a boundary between the regions of higher (for lower iteration numbers) and lower (for the higher iteration numbers) steepness of the cost function can be detected. The difference in steepness causes certain values of the step size to be a better choice for certain applications. For example, if the expected number of iterations is lower than 5, one should chose the step size with the steepest graph in that region (i.e., μ=0.07) since this step size yields the estimate in lowest number of iterations. On the other hand, different step size may be more suitable if an application requires larger number of iterations. If the number of iterations exceeds 15, the optimal choice represents the step size μ=0.11.

Although the analysis presented above is based on the specific case of the rotational quaternion defined by the specific angle of rotation $\alpha =45^{\circ }$, the same behavior is observable for different angles of rotation presented in Figure 2b–d. By increasing the angle of rotation, the distance between the true and initial rotational quaternion in quaternion space increases. Because of that, the initial value of the cost function increases with the angle α. Moreover, in the 30 iterations presented in Figure 2, different final values of the cost function are achieved indicating the difference in accuracy of the estimate. This behavior is characteristic for iterative algorithms. However, notice that the step sizes yielding the fastest convergence for the lower and the higher iteration numbers remain μ=0.07 and μ=0.11, respectively. Since the number of iterations in attitude estimation based on the presented algorithm is expected to be small, the step size is set to μ=0.07.

### 4.2. Termination of Iterative Process

In the previous examples, the step size and the cost function are analyzed with the fixed number of iterations set to 30, although, in the steady state, the number of iterations used for attitude estimation is expected to be much lower. In some publications, a single iteration is assumed to be sufficient [30,34]. However, this hypothesis is difficult to prove without setting a figure of merit. Here, we analyze different methods and approaches for the termination of the gradient descent iterative process. In general, the process can be terminated based on the number of iterations or desired accuracy. These two criteria represent opposing requirements.

The first and the simplest approach for the termination of the iterative process is setting the maximum number of iterations $N_{max}$. In perfect noiseless conditions, as the number of iterations approaches infinity, an estimate approaches the ground truth. Thus, setting the finite number of iterations leads to the loss of accuracy. On the other hand, aiming for the highest accuracy requires large (theoretically infinite) number of iterations, which significantly increases the processing time. If the time constraint is the limiting factor of the application, one can reduce the duration of the gradient descent algorithm by limiting the maximum number of iterations by setting $N_{max}$. The value of $N_{max}$ highly depends on the processing power of the targeted processing unit.

If, on the other hand, the desired accuracy of the estimate is reached, the iterative process should be terminated. However, a figure of merit is needed to properly set the termination criterion. In ideal lossless conditions, the termination criterion can be set based on the value of the cost function. Recall that, if the step size is set correctly, the value of the cost function monotonically decreases with each iteration. If the value of the cost function J(q) drops below some a priori defined value $J_{max}$, the desired accuracy is reached and the iterative process can be terminated. Thus, $J_{max}$ represents the user defined positive real number that sets the highest acceptable error and thus the desired accuracy of the estimate. The lower the $J_{max}$, the higher the accuracy. Unfortunately, such a criterion is impractical in real world applications. The reason for that lies in the fact that the measurements of physical quantities based on which the rotational quaternion is estimated are always affected by noise. In the presence of noise, the gradient descent algorithm is still able to find the rotational quaternion that minimizes the cost function, however, the value of the cost function at its minimum is no longer equal to zero, causing it to be practically impossible to reach the desired accuracy by setting $J_{max}$. However, as the estimate approaches the ground truth, the difference between the values of the cost function in current and previous iteration becomes smaller. Eventually, at the minimum of the cost function, the difference becomes equal to zero. Thus, the termination criterion can be set based on parameter $\Delta J_{max}$. Such a criterion is reported in [26]. Here, we proposed yet another criterion based on the gradient of the cost function $\nabla J(q_{k})$ defined by (16). Indeed, calculating the difference between two neighboring points of the cost function is very similar to finding its derivative. This information is also contained in the gradient itself, which is calculated in each iteration of the gradient descent algorithm. If the minimum of the cost function is reached, $\nabla J(q_{k})$ becomes a zero vector. Thus, to determine the accuracy of the gradient descent algorithm, we introduce the scalar representation of the gradient *G* defined as:

(22) $$G={\left(\nabla J(q)\right)}^{T}\nabla J\left(q\right).$$

The iterative process can be terminated in the *k*-th iteration if $G_{k}<G_{max}$, here $G_{max}$ is an arbitrarily chosen user-defined positive real number. Similar to $\Delta J_{max}$, by decreasing the value of $G_{max}$, the accuracy of the quaternion estimation increases at the cost of the number of iterations.

**Figure 3:**
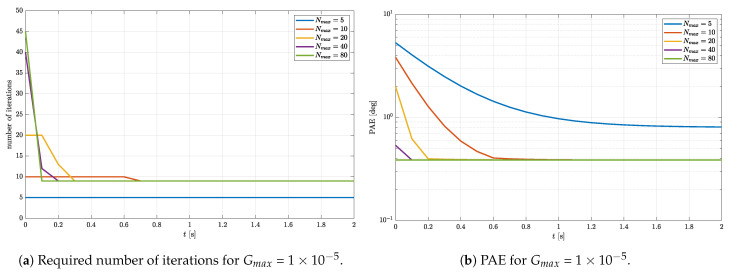
Required number of iterations and PAE of the estimated rotational quaternion (μ=0.7).

To gain a better understanding of how $N_{max}$ and $G_{max}$ affect the quality of the rotational quaternion estimation, another simulation is performed. In this simulation, it is assumed that an object rotates with the constant angular speed of 20 deg/s around an arbitrary chosen vector of rotation $n={\left[\begin{array}{ccc} 1 & -1 & 0 \end{array}\right]}^{T}$. The initial angle of rotation around the vector n is chosen to be $\alpha_{0}=45^{\circ }$. Based on the provided data, the true rotational quaternion is calculated for each time step. The time period between the calculated quaternion is chosen to be 0.1 s. The true quaternion is then used to calculate the body frame gravitational acceleration and magnetic field that are passed to the gradient descent algorithm as the input arguments at each time step. The initial quaternion for the very first iterative process was chosen to be $q_{0}={\left[\begin{array}{cccc} 1 & 0 & 0 & 0 \end{array}\right]}^{T}$. However, the rotational quaternion estimated in every other iterative process is passed as the initial quaternion for the next iterative process. This is one of the initial quaternion propagation methods that will be discussed in detail further on. In this simulation, the number of iterations and PAE were monitored with $G_{max}$ fixed to $1\times 10^{-5}$ for different $N_{max}$ in the range from 5 to 80. The step size is chosen to be μ=0.07. The simulation results are shown in Figure 3.

In Figure 3a, one can observe that $N_{max}$ has a dominant effect on the duration of the transient, i.e., the number of samples of the gravitational acceleration and the magnetic field needed for the algorithm to converge. Indeed, the higher the $N_{max}$, the quicker the steady state is reached. In a steady state, the number of iterations required for reaching the accuracy defined by $G_{max}$ = $1\times 10^{-5}$ is equal to 9 for every $N_{max}$, except for $N_{max}=5$. Notice that, for $N_{max}=5$, the number of iterations is maxed out during the whole simulation period. This indicates that five iterations are simply not enough for reaching the required accuracy. This is also evident in Figure 3b. Notice that, in a steady state, the same PAE is reached for each $N_{max}$ except for $N_{max}=5$. The PAE is greater for the $N_{max}=5$, therefore indicating an insufficient number of iterations. Furthermore, one can notice that, during the transient, the number of iterations is maxed out for each $N_{max}$ except for $N_{max}=80$ for which the steady state is reached in only 0.1 s, indicating a single sample of gravitational acceleration and magnetic field.

**Figure 4:**
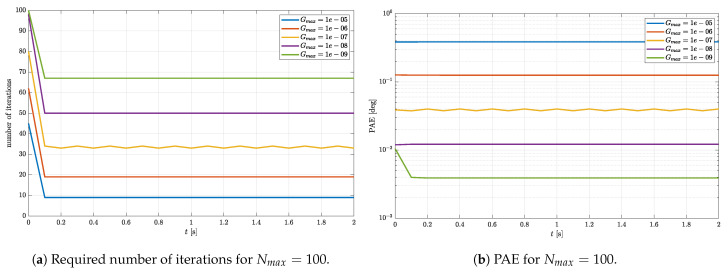
Required number of iterations and PAE of the estimated rotational quaternion μ=0.7.

The same simulation is repeated with $N_{max}$ set to the fixed value of 100, while $G_{max}$ is varied in the range from $1\times 10^{-9}$ to $1\times 10^{-5}$. Since the number of iterations is set to a relatively high value, a quick convergence is expected for each $G_{max}$. This is confirmed by the results shown in Figure 4a. However, the number of iterations in a steady state is not equal for every $G_{max}$. The lower the $G_{max}$, the higher the number of iterations required for reaching the desired accuracy. This is evident from Figure 4b as well. If $G_{max}$ is smaller, the small PAE is reached in a steady state. This is a clear confirmation that parameters *G* defined in (22) and $G_{max}$ represent a valid figure of merit and termination criterion.

**Figure 5:**
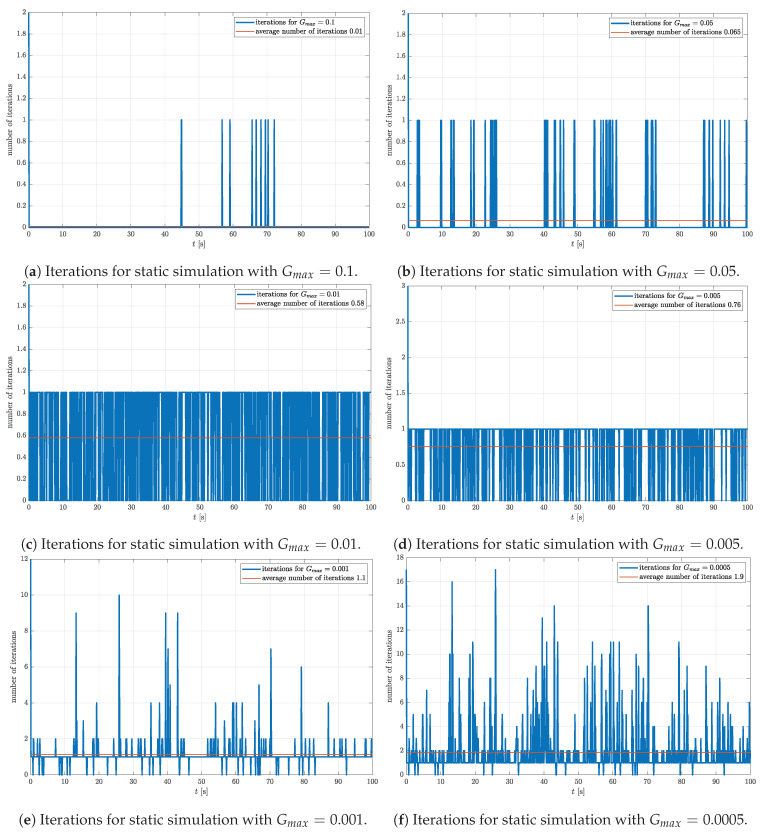
Number of gradient descent iterations required for estimation of rotation quaternion based on a previous estimate in static condition for six values of $G_{max}$ in range from $1\times 10^{-1}$ to $5\times 10^{-4}$ and for μ=0.7.

The simulation results presented above show that, if the $G_{max}$ is too high, the accuracy of the estimate is too low. On the other hand, if the $G_{max}$ is too low, the desired accuracy may be too high with respect to $N_{max}$. Increasing $N_{max}$ leads to a larger number of iterations. If a targeted processing unit is capable of processing a large number of iterations in a given amount of time, this is a perfectly viable option in lossless conditions. However, in the presence of noise, a higher number of iterations does not necessarily mean higher accuracy. Thus, setting $G_{max}$ too low may lead to wasting computational resources. Naturally, there is an optimal range of values of $G_{max}$, which should be determined using simulations or experiments. The optimal $G_{max}$ minimizes the number of iterations required in a steady state. Here, we propose the method to determine the optimal $G_{max}$. The method is based on static simulation with noise added to the gravitational acceleration and magnetic field. The assumed attitude is represented by the rotational quaternion defined by the vector and angle of rotation, $n={\left[\begin{array}{ccc} 1 & -1 & 0 \end{array}\right]}^{T}$ and $\alpha_{0}=45^{\circ }$, respectively. The initial quaternion is again chosen to be $q_{0}={\left[\begin{array}{cccc} 1 & 0 & 0 & 0 \end{array}\right]}^{T}$. The rotation quaternion estimated for each sample is passed as the initial quaternion in the next iterative process. In a steady state, without the presence of noise, the previously estimated quaternion is the same as the current quaternion since the simulation is static. Consequently, the number of iterations per iterative process is expected to drop to zero in a steady state, regardless of $G_{max}$. However, in the presence of noise, zero iterations per iterative process means that the selected value of $G_{max}$ is not optimal. In that case, higher accuracy can be achieved by decreasing the value of $G_{max}$. $G_{max}$ should be decreased until the number of iterations starts to increase. Decreasing the $G_{max}$ even further leads to the increase in the number of iterations without gaining accuracy. Since the object is static, the additional iterations appear solely due to the sensor noise. This conclusion is supported by the simulation results shown in Figure 5 representing the number of iterations for $G_{max}$ in a range from $1\times 10^{-1}$ to $5\times 10^{-4}$.

All the graphs in Figure 5 have the highest number of iterations around t=0 s caused by the difference between the true and the initial quaternion, which quickly vanishes. This effect is detailed in the previous section. Figure 5a,b represent results with the highest $G_{max}$. The average number of iterations over the simulation period of 100 s is below 0.1. Based on the presented results, one can conclude that the chosen values of $G_{max}$ are too high and that the accuracy of the estimate can be further improved by decreasing the value of $G_{max}$. On the other hand, Figure 5e,f represent the opposite scenario. Although the average number of iterations is not unreasonably high (below 2), at some time steps, a significantly larger number of iterations is needed to achieve the desired accuracy. This is an indication that the selected $G_{max}$ is too low, which results in a noisy estimate. Thus, one concludes that optimal $G_{max}$ yields the average number of iterations around the midpoint of the 0–1 range. Figure 5c,d represent the results obtained for $G_{max}$ equal to $1\times 10^{-2}$ and $5\times 10^{-3}$, respectively. In these cases, the number of iterations does not exceed 1 at any time step. Furthermore, notice that, in both cases, the average number of iterations is closer to 0.5. Thus, the $G_{max}$ is fixed to $1\times 10^{-2}$ for the next simulations in which we examine different methods of the initial quaternion propagation.

### 4.3. Propagation of Initial Quaternion

There are several approaches to selecting the initial quaternion $q_{0}$ for the gradient descent algorithm. Here, we present and examine three different propagation methods. In propagation method 1, a fixed arbitrarily selected rotation quaternion is used for each sample/iterative process. Since it is not possible to know the attitude of an object a priori, each rotational quaternion selected as an initial quaternion represents a reasonable guess. In propagation method 2, the rotational quaternion estimated in current iterative process is used as an initial quaternion of the next iterative process. This propagation method was briefly introduced in the sections above. Here, we use the assumption that the attitude of an object cannot change instantaneously (or cannot change much in a short period of time). Thus, the previously estimated quaternion is expected to be closer to the current quaternion, which reduces the number of iterations in comparison with the previous method. Finally, propagation method 3 uses an attitude estimate and information about the angular rate at the current time step to predict the rotational quaternion at the next time step. This prediction is then used as an initial quaternion in the next iterative process.

Unlike the other gradient descent based attitude estimation methods reported in the literature [30,34], which fix the number of iterations to 1, adaptively changing the step size with respect to the measured angular velocity, the iterative process here is performed with an initial quaternion closest to the true quaternion until the desired accuracy defined by $G_{max}$ = $1\times 10^{-2}$ is reached. This method is expected to yield the lowest number of iterations. All three propagation methods are implemented as a part of the complementary filter. The complementary filter fuses the quaternions estimated using the gradient descent algorithm (based on the gravitational acceleration and magnetic field data) and quaternions estimated from the angular velocity. Here, the complementary filter gain is set to K=0.5, providing the same weight to each of the quaternion estimation methods. The value of the parameter *K* is not optimized, as it is not part of the gradient descent algorithm per se.

**Figure 6:**
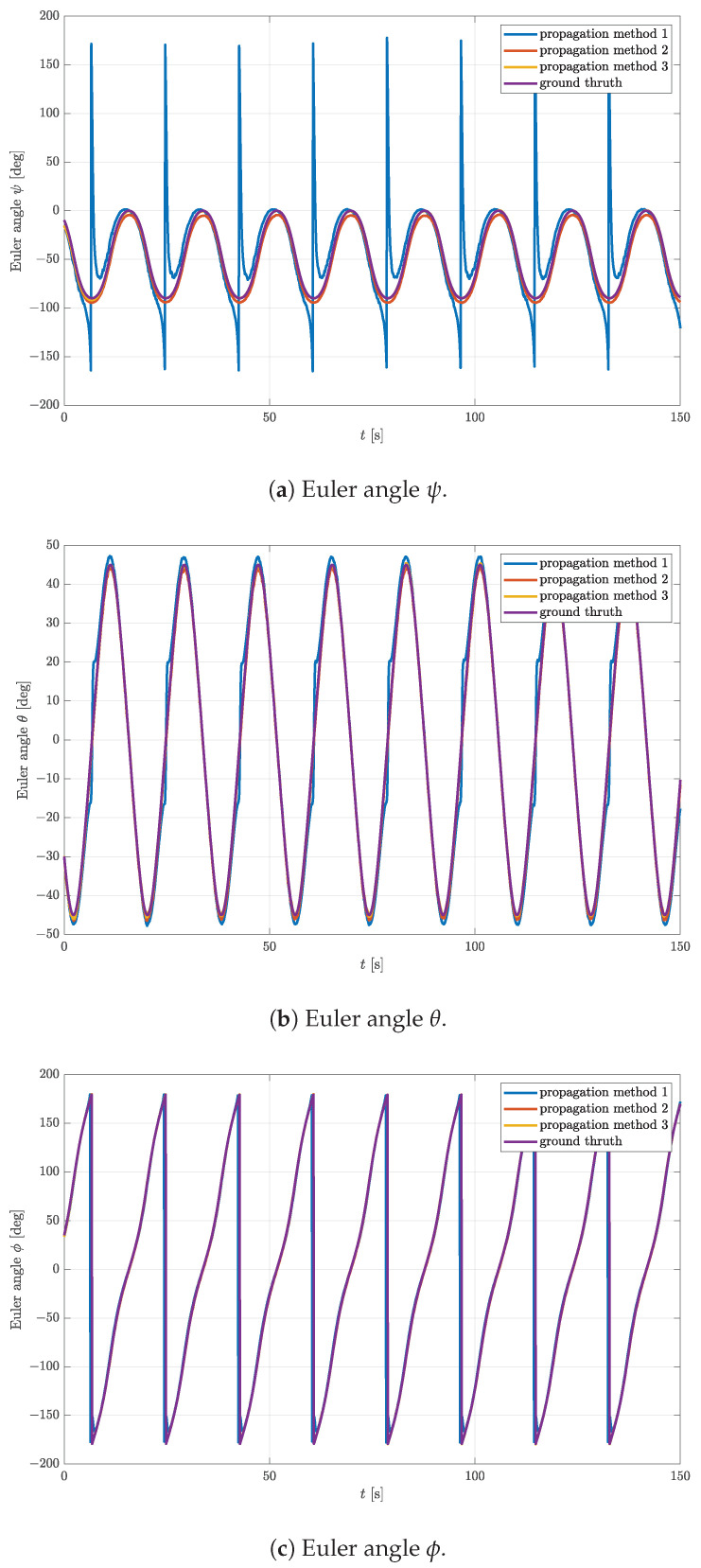
Comparison of estimated Euler angles using different propagation methods and complementary filter with parameters μ=0.7, $N_{max}=20$, $G_{max}$ = $1\times 10^{-2}$, and K=0.5.

In this simulation, it is assumed again that an object rotates with the constant angular speed of 20 deg/s around an arbitrary chosen vector of rotation $n={\left[\begin{array}{ccc} 1 & -1 & 0 \end{array}\right]}^{T}$. The initial quaternion and angle of rotation around the vector n are chosen to be $q_{0}={\left[\begin{array}{cccc} 1 & 0 & 0 & 0 \end{array}\right]}^{T}$ and $\alpha_{0}=45^{\circ }$, respectively. For each time step, the true rotational quaternion is calculated, which is then used to calculate the body frame gravitational acceleration and magnetic field. The angular velocity vector remains the same in both frames since it lies on the axis of rotation. Corresponding noise is added to each physical quantity and passed to the complementary filter implemented in MathWorks Matlab with the sample time of 0.1 s. The rotational quaternions estimated in each time step using all three initial quaternion propagation methods are converted in Euler angles and shown in Figure 6. Ppropagation methods 2 and 3 show excellent results with respect to the ground truth. Propagation method 1, however, had difficulties estimating the rotational quaternion even with the maximum iteration number $N_{max}=20$, which is especially manifested in the plot of Euler angle ψ in Figure 6a.

**Figure 7:**
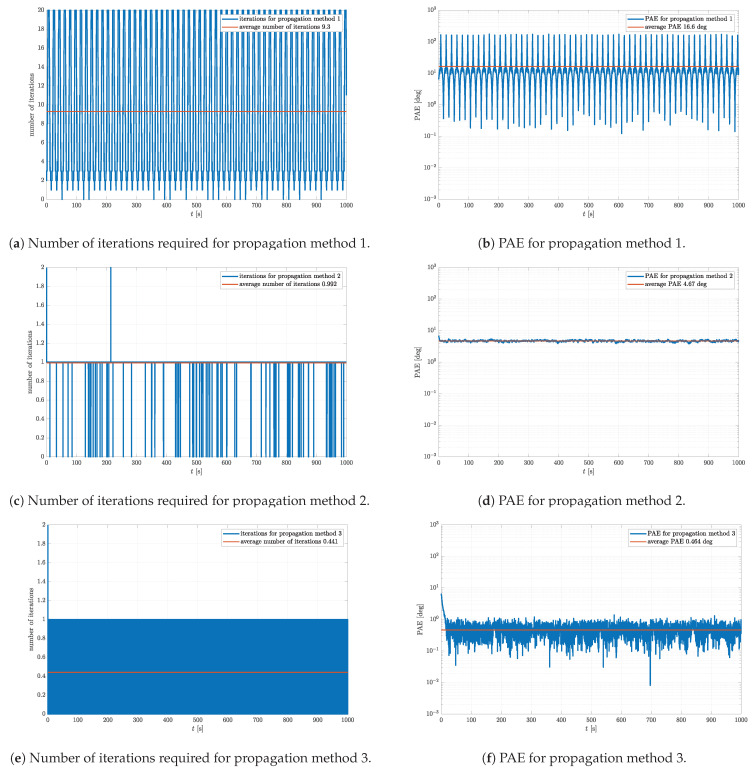
Comparison of number of iterations and PAE for different propagation methods and complementary filters with parameters μ=0.7, $N_{max}=20$, $G_{max}$ = $1\times 10^{-2}$, and K=0.5.

At some specific time steps, the initial quaternion is simply not good enough for the gradient descent to converge, which manifests in periodic spikes. On the other hand, Euler angles θ and ϕ are estimated reasonably well even with propagation method 1. The number of required iterations and PAE for the same simulation are shown in Figure 7.

Figure 7 clearly shows the difference in performance between the three propagation methods. It is clear that the iteration number is maxed out at exactly the same time steps at which the Euler angle ψ is incorrectly estimated using propagation method 1 (Figure 7a). This is evident even from the PAE in Figure 7b. This indicates that propagation method 1 is non-optimal, which is also confirmed by a relatively large average number of iterations of 9.3 and PAE of 16.6${}^{\circ }$. Propagation method 2 yields much more reasonable results with respect to the number of iterations and the PAE shown in Figure 7c,d, respectively. Notice that the average number of iterations is very close but is still below 1. This means that the iterative process is not always needed for the rotational quaternion estimation. This can also be observed at the time steps at which the number of iterations is equal to zero. It is interesting that the noise added to the simulated data actually helps to bring the initial quaternion closer to the optimal estimate, which leads to the desired accuracy without applying a single iteration of a gradient descent algorithm. This effect is very similar to the process of intentionally applying noise known as dithering [35]. Furthermore, notice that the average PAE of 4.67${}^{\circ }$ is drastically improved with respect to propagation method 1. As for propagation method 3, the results of which are shown in Figure 7e,f, it evidently yields the best results. The average PAE is over an order of magnitude lower with respect to propagation method 2, i.e., 0.464${}^{\circ }$. Moreover, notice that the noise floor of the PAE is reached. This confirms that $G_{max}$ defining the accuracy of the estimate is correctly set. The improvement is clearly visible in the average number of iterations, which is dropped to 0.441. Thus, one can conclude that the attitude estimation algorithm mostly relies upon the angular velocity data, which for MPU9250 have the lowest noise level of the three. This noise level is decreased even further through the numerical integration characteristic for the estimation of the rotational quaternion from angular velocity. Only now and then is the estimate automatically corrected using the gradient descent algorithm if $G>G_{max}$.

## 5. Experimental Results

**Figure 8:**
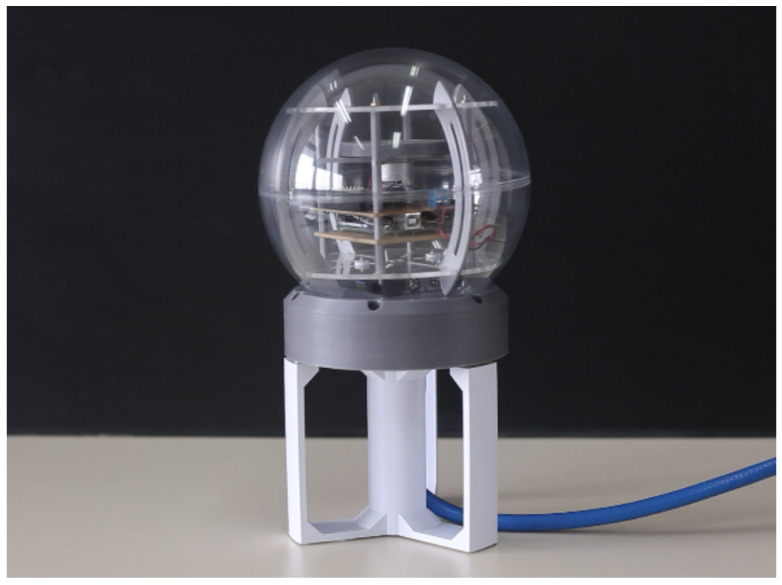
Photograph of the ADCS model used for experimental verification of the presented attitude estimation algorithm based on gradient descent and complementary filter.

The presented attitude estimation method is based on the gradient descent algorithm, complementary filter, and propagation method 3 and is experimentally tested using the model of the satellite attitude determination and control system (ADCS) shown in Figure 8. The model was developed for educational purposes at the University of Zagreb, Faculty of Electrical Engineering and Computing, Department of Communication and Space Technologies.

**Figure 9:**
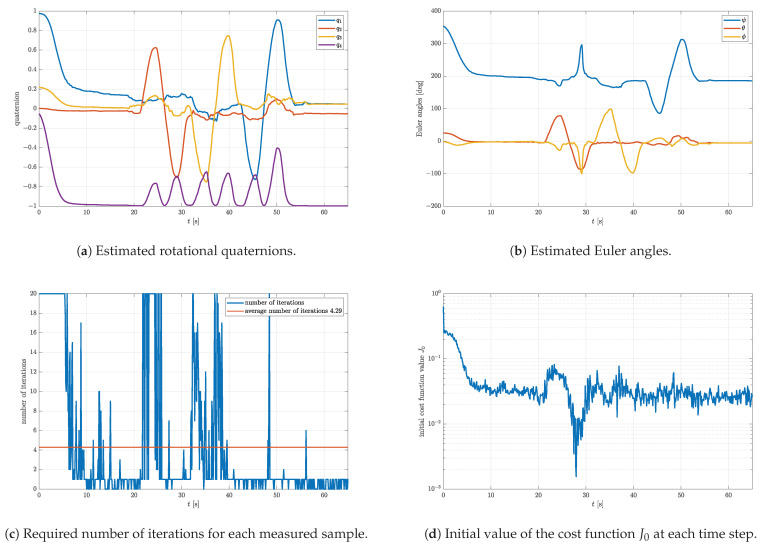
Experimental results obtained with parameters μ=0.7, $q_{0}={\left[\begin{array}{cccc} 1 & 0 & 0 & 0 \end{array}\right]}^{T}$, $N_{max}=20$, $G_{max}$ = $1\times 10^{-2}$, and K=0.5.

The ADCS model consists of a microcontroller, Bluetooth interface, reaction wheel (not used in this experiment), and the MPU9250 IMU with a 3-axis accelerometer, magnetometer, and gyroscope. The nine measurements were sampled with the sampling frequency of 10 Hz, wirelessly transferred to a computer and then used as the input arguments of the implemented attitude estimation algorithm. For the experimental purposes, the model is rotated for approximately $\pm 90^{\circ }$ around three orthogonal axes by hand. The initial quaternion for the first sample was chosen to be $q_{0}={\left[\begin{array}{cccc} 1 & 0 & 0 & 0 \end{array}\right]}^{T}$. However, the ADCS model was initially rotated for approximately 180${}^{\circ }$ around the *z*-axis, which represents the farthest point in quaternion space with respect to the initial quaternion and the worst case scenario. Please recall the results shown in Figure 2. Notice that the final value of the cost function shown in Figure 2d is indeed the highest compared to the other angles α for the same step size and number of iterations. The estimated quaternions and corresponding Euler angles are shown in Figure 9 together with the required number of iterations per sample and initial value of the cost function at each time step.

Figure 9 shows the perfect example of a real time scenario. In Figure 9b, the rotations for approximately $\pm 90^{\circ }$ around all three orthogonal axes, representing the Euler angles ψ, θ, and ϕ, are clearly visible. Notice that the average number of iterations is significantly higher than simulated (Figure 9c). The reason for that is the presence of other imperfections and influences on the IMU sensors. Recall that, for example, the accelerometers measure not only the gravitational acceleration. Rotating the model by itself may introduce additional acceleration and may interfere with the measurement of gravitational acceleration. Likewise, the magnetometers suffer from hard and soft iron effects caused by different magnetic materials present in the vicinity of the sensor. Although gyroscopes show the best performances of the three in terms of noise, they are not immune to issues such as bias stability and angle random walk, which may significantly affect attitude estimation. All these effects are difficult to model and account for in simulations. Thus, the experimental validation of the chosen gradient descent parameter set is of the highest importance. It is fairly straightforward to show that these secondary effects cause a larger number of iterations by plotting the initial value of the cost function at each time step, as shown in Figure 9d. Since propagation method 3 is used to propagate the initial quaternion, it is expected that the initial value of the cost function at each time step monotonically decreases down to its minimum. For the first sample of the simulation, the initial value of the cost function is expected to be at its maximum due to the difference between the true and guessed initial quaternion. This is indeed the case. However, in the time interval of 20–40 s, a sudden increase in the initial cost function value can be observed. This increase is caused by the secondary effects, which manifest as an undesired signal superposed to the measurements. The consequence of the secondary effect is the lower accuracy of the estimated rotational quaternion. Since the accuracy of the attitude estimation is lowered, it is justified to slightly increase the value of the parameter $G_{max}$.

**Figure 10:**
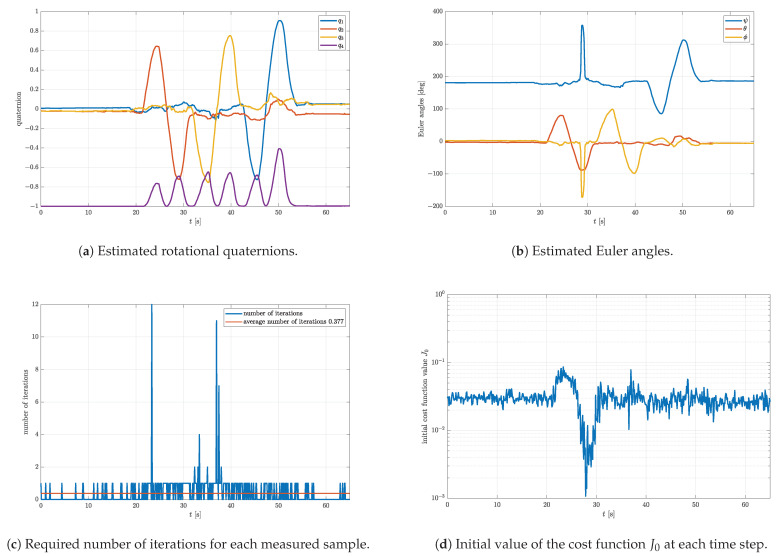
Experimental results obtained with parameters μ=0.7, $q_{0}={\left[\begin{array}{cccc} 0 & 0 & 0 & 1 \end{array}\right]}^{T}$, $N_{max}=20$, $G_{max}$ = $3\times 10^{-2}$, and K=0.5.

**Figure 11:**
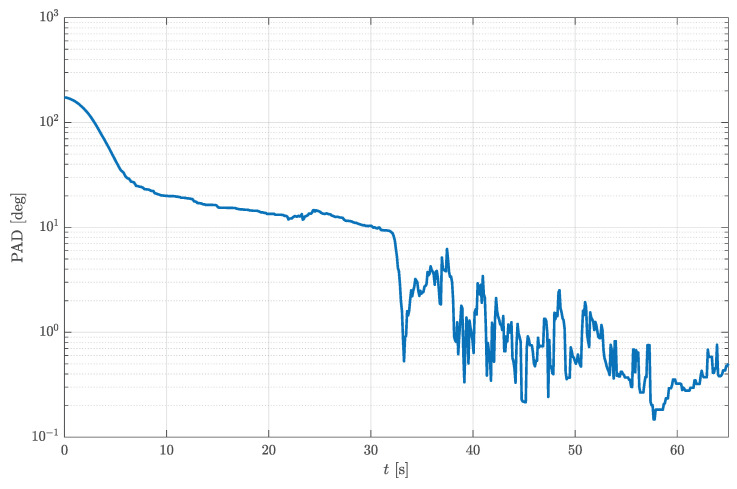
PAD between the estimated rotational quaternions for both sets of parameters.

To prove that the initial value of the cost function is highest at the beginning of the measurement (t∈[0,10] s) due to the difference between the initial and true quaternion, the estimation is repeated with the initial quaternion set to $q_{0}={\left[\begin{array}{cccc} 0 & 0 & 0 & 1 \end{array}\right]}^{T}$. This new initial quaternion represents the rotation for $180^{\circ }$ around *z*-axis, which approximately describes the true initial attitude of the ADCS model. Likewise, the value of $G_{max}$ was increased to $3\times 10^{-2}$. The results are presented in Figure 10. Since the initial quaternion is very close to the true rotational quaternion, it is clear that the transient is fully eliminated, which manifests through constant Euler angles (Figure 10b), a low number of iterations (Figure 10c), and a constant initial value of the cost function (Figure 10d) for t∈[0,10] s. As expected, the sudden increase in the initial cost function value is still present in the time interval of 20–40 s. Nevertheless, the number of iterations in the same period is not maxed out due to the higher $G_{max}$. Furthermore, the average number of iterations is significantly lower (only 0.377) compared to the previous estimation (Figure 9c). This significantly unloads the targeted processing unit and lowers the processing power requirement. Most of the time not a single iteration of the gradient descent algorithm is needed. During these periods, the estimation of the rotational quaternion relies upon the gyroscope only. Sometimes, however, if the measurement is corrupted or the gyroscope bias accumulated, the value of the cost function increases, which automatically activates the gradient descent algorithm and corrects the estimate. As shown in Figure 9c, the number of gradient descent iterations may be significantly higher than one. This demonstrates the importance of the valid termination criterion. It also leads to the conclusion that it is not sufficient to limit the number of iterations to one. Increasing $G_{max}$ from $1\times 10^{-2}$ to $3\times 10^{-2}$ does not substantially affect the quality of the attitude estimation, as shown in Figure 11. The graph shown in Figure 11 represents the principal angle deviation (PAD) between the estimated rotational quaternions for both parameter sets. It starts at higher values, caused by the transient of the first estimation, but eventually drops to very low values with a minimum below $0.2^{\circ }$.

## 6. Conclusions

In this paper, a comprehensive approach to the optimization of the gradient descent parameters with an application in the estimation of rotational quaternions is presented. The goal of the optimization was to reduce the computational complexity of the algorithm by minimizing the required number of iterations while maintaining the accuracy of the estimation. Most of the gradient descent attitude estimation methods limit the number of iterations to one. Here, however, we showed that such a low number of iterations may not be sufficient for achieving the desired accuracy under the influence of different sensor imperfections and external factors. Nevertheless, using the proposed approach, the average number of iterations may be much lower than one. The number of iterations is determined automatically based on the presented termination criterion. The optimized gradient descent algorithm was used as a part of complementary filter fusing the measurements acquired from low cost MPU9250 IMU sensors. The low average number of iterations and simplicity of implementation causes the proposed method to be ideal for low-power applications. The same approach to attitude estimation based on the gradient descent algorithm and its optimization are suitable and may be used as a part of more demanding attitude estimation algorithms employing Kalman filtering, which will be the subject of our future research efforts.
